# Mitochondrial DNA disorders in neuromuscular diseases in diverse populations

**DOI:** 10.1002/acn3.52141

**Published:** 2024-08-02

**Authors:** Fei Gao, Katherine R Schon, Jana Vandrovcova, Özlem Yayıcı Köken, Sharika Raga, Kireshnee Naidu, Maryke Schoonen, Nimita Rani, Pedro Tomaselli, Dipti Baskar, Musambo Kapapa, Ipek Polat, Lindsay A Wilson, Kumarasamy Thangaraj, Uluç Yiş, Bevinahalli N Nandeesh, David Bearden, Michelle Kvalsund, Franclo Henning, Seena Vengalil, Atchayaram Nalini, Claudia F. R. Sobreira, Wilson Marques, Haluk Topoloğlu, Michael G Hanna, Sireesha Yareeda, Venugopalan Y Vishnu, Francois H van der Westhuizen, Izelle Smuts, Surita Meldau, Jo Wilmshurst, Büşranur Çavdarlı, Jeannine Heckmann, Patrick F Chinnery, Rita Horvath

**Affiliations:** ^1^ Department of Clinical Neurosciences University of Cambridge Cambridge UK; ^2^ MRC Mitochondrial Biology Unit University of Cambridge Cambridge UK; ^3^ Academic Department of Medical Genetics University of Cambridge Cambridge UK; ^4^ UCL Queen Square Institute of Neurology, University College London London UK; ^5^ Department of Pediatric Neurology, Faculty of Medicine Akdeniz University Antalya Turkey; ^6^ Division of Paediatric Neurology, Department of Paediatrics and Child Health Red Cross War Memorial Children's Hospital Cape Town South Africa; ^7^ Division of Neurology Neuroscience Institute, University of Cape Town Cape Town South Africa; ^8^ Neurology Research Group, Division of Neurology, Department of Medicine University of Cape Town Cape Town South Africa; ^9^ Division of Neurology, Department of Medicine, Faculty of Medicine and Health Sciences Stellenbosch University Cape Town South Africa; ^10^ Focus Area for Human Metabolomics North‐West University Potchefstroom South Africa; ^11^ Department of Neurology All India Institute of Medical Sciences (AIIMS) Delhi India; ^12^ Department of Neuroscience Clinical Hospital of Ribeirão Preto Medical School of, University of São Paulo São Paulo Brazil; ^13^ Department of Neurology National Institute of Mental Health & Neurosciences (NIMHANS) Bengaluru India; ^14^ Department of Physiotherapy, School of Health Sciences University of Zambia, University Teaching Hospital Neurology Research Office Lusaka Zambia; ^15^ Izmir Biomedicine and Genome Center (IBG) Izmir Turkey; ^16^ Izmir International Biomedicine and Genome Institute Dokuz Eylul University Izmir Turkey; ^17^ CSIR—Centre for Cellular and Molecular Biology (CCMB) Hyderabad Telangana India; ^18^ Pediatric Neurology Department, Faculty of Medicine Dokuz Eylül University Izmir Turkey; ^19^ Department of Neuropathology National Institute of Mental Health & Neurosciences (NIMHANS) Bengaluru India; ^20^ Department of Neurology University of Rochester Rochester New York USA; ^21^ Department of Internal Medicine University of Zambia School of Medicine Lusaka Zambia; ^22^ Yeditepe University Hospitals Istanbul Turkey; ^23^ Nizam's Institute of Medical Sciences (NIMS) Hyderabad Telangana India; ^24^ Department of Paediatrics, Steve Biko Academic Hospital University of Pretoria Pretoria South Africa; ^25^ Division of Chemical Pathology, Department of Pathology University of Cape Town Medical Faculty Cape Town South Africa; ^26^ National Health Laboratory Service Cape Town South Africa; ^27^ Department of Medical Genetics Ankara Bilkent City Hospital Ankara Turkey; ^28^ Faculty of Medicine, Department of Medical Genetics Ankara Yıldırım Beyazıt University Ankara Turkey; ^29^ Neuroscience Institute, University of Cape Town Cape Town South Africa; ^30^ Division of Neurology, Department of Medicine Groote Schuur Hospital Cape Town South Africa

## Abstract

Neuromuscular features are common in mitochondrial DNA (mtDNA) disorders. The genetic architecture of mtDNA disorders in diverse populations is poorly understood. We analysed mtDNA variants from whole‐exome sequencing data in neuromuscular patients from South Africa, Brazil, India, Turkey and Zambia. In 998 individuals, there were two definite diagnoses, two possible diagnoses and eight secondary findings. Surprisingly, common pathogenic mtDNA variants found in people of European ancestry were very rare. Whole‐exome or ‐genome sequencing from undiagnosed patients with neuromuscular symptoms should be re‐analysed for mtDNA variants, but the landscape of pathogenic mtDNA variants differs around the world.

## Introduction

Neuromuscular phenotypes are frequent in multi‐system mitochondrial disorders^1^ and can occur in isolation, for example, isolated mitochondrial myopathy with the m.3243A>G ‘MELAS’ pathogenic variant,^2^ and isolated peripheral neuropathy with pathogenic variants in *MT‐ATP6*.^3^ A pathogenic mtDNA variant was identified in 3.8% of participants with peripheral neuropathy in the RD‐Connect study.^4^ However, there are no data on the diagnostic yield of mtDNA analysis in large neuromuscular disease cohorts to date.

People's mtDNA genetic background (haplogroup) reflects their maternal ancestry. There are three lineages—N (‘Eurasian’), L (‘African’) and M (‘Asian’) with multiple haplogroups in each.^5^ The haplogroup can affect the penetrance of mtDNA pathogenic variants, for example the m.11778A>G and m.14484T>C variants in Leber Hereditary Optic Neuropathy (LHON) show higher penetrance on the J haplogroup.^6^

Little is known about genetic architecture of mtDNA disorders in diverse populations. A general lack of common syndrome‐associated mutations was reported in a South African cohort of paediatric mitochondrial disease patients.^7^ However, another study from South Africa identified m.3243A>G, LHON and single large‐scale mtDNA deletions as the commonest causes of mitochondrial disorders.^8^ European individuals are highly over‐represented in commonly used genetic databases and have dominated our understanding of genetic epidemiology. For example, 70% of individuals in gnomAD v3.1 have haplogroups of N lineages with 25% L lineages and 5% M lineages.^9^

Off‐target whole‐exome sequencing (WES) reads can be used to reliably determine the mtDNA sequence, providing there is adequate read depth,^10^ however, this is not included in most diagnostic tests or research studies. Previous research suggests that mtDNA analysis provides a modest diagnostic uplift in patients with neurological disorders.^11^, ^12^, ^13^

The International Centre for Genomic Medicine in Neuromuscular Disorders is a remote transcontinental partnership which aims to study the genetic architecture of neuromuscular diseases in diverse populations.^14^ Families with neuromuscular disorders are recruited from India, Brazil, South Africa, Turkey and Zambia. We present results of mtDNA analysis of WES data from 998 probands with neuromuscular disorders from four continents. We explore the diagnostic yield and genetic architecture of mtDNA disorders.

## Methods

Families with suspected inherited neuromuscular disorders were recruited through neurology and paediatric neurology clinics at tertiary referral centres in India, Brazil, South Africa, Turkey and Zambia using linked ethically approved local and national studies.^14^ Diagnostic categories were assigned by the recruiting neurologist or paediatric neurologist. Mitochondrial disorders were suspected in individuals with multi‐system disorders, usually affecting the central nervous system and/or musculature, or with investigation results suggestive of mitochondrial disorders such as raised serum lactate. Whole‐exome sequencing was performed in 1122 participants on DNA extracted from blood. Libraries were generated in four centres using different protocols^14^ (see Appendix S1). Samples were sequenced on the Illumina Novaseq 6000 platform to a minimum of 30× coverage.

The MToolBox pipeline^15^ was used to call mtDNA variants and assign haplogroups. Variants with depth <10 or quality score <30 were excluded. Variants were annotated with population frequencies (gnomAD v3.1^9^) and MitoMap^16^ status. MitoMap ‘confirmed’ variants (with strong evidence of pathogenicity) and MitoMap ‘reported’ variants (considered possibly pathogenic) with allele frequency <0.05 were reviewed by mitochondrial clinicians. To identify novel, potentially causative variants, the data were filtered to identify nonsynonymous variants in protein‐coding genes and variants in tRNA genes with a heteroplasmy fraction of 10–90% which had a population frequency <1 in 50,000 in gnomAD v3.1.

The phenotypic fit and heteroplasmy level were considered. American College of Medical Genetics guidelines for mtDNA variant interpretation^17^ were applied. Research reports were sent to recruiting clinicians. Additional investigations were undertaken when requested by the local clinical teams. In the family with the m.9984G>A variant in *MT‐CO3*, whole mitochondrial‐genome sequencing was undertaken in blood, urine and buccal samples in the child and mother. In the family with the m.8993T>G variant in *MT‐ATP6*, additional testing was done by restriction fragment length polymorphism testing in DNA extracted from blood in the child and in DNA extracted from blood and urine in the mother.

## Results

### Description of cohort

**Figure 1:**
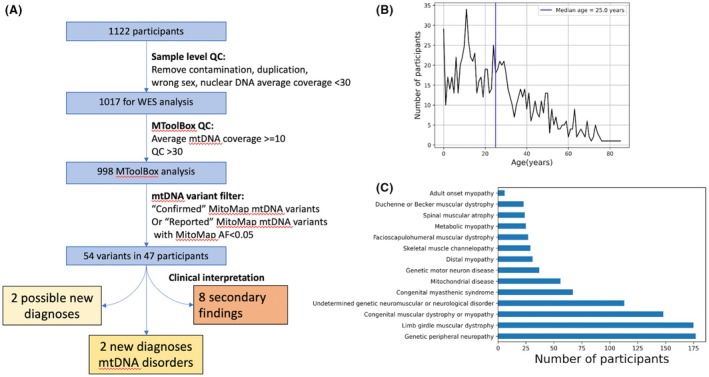
(A) Flow diagram summarising filtering steps applied to 1122 participants from diverse populations who had exome sequencing for investigation of neuromuscular disorders between 2021 and 2023. (B) Number of participants by age. Age ranges from infancy to 86 years with median age of 25 years. (C) Number of probands assigned to each of 14 diagnostic categories. The commonest categories were genetic peripheral neuropathy, limb‐girdle muscular dystrophy and congenital muscular dystrophy or myopathy.

**Figure 2:**
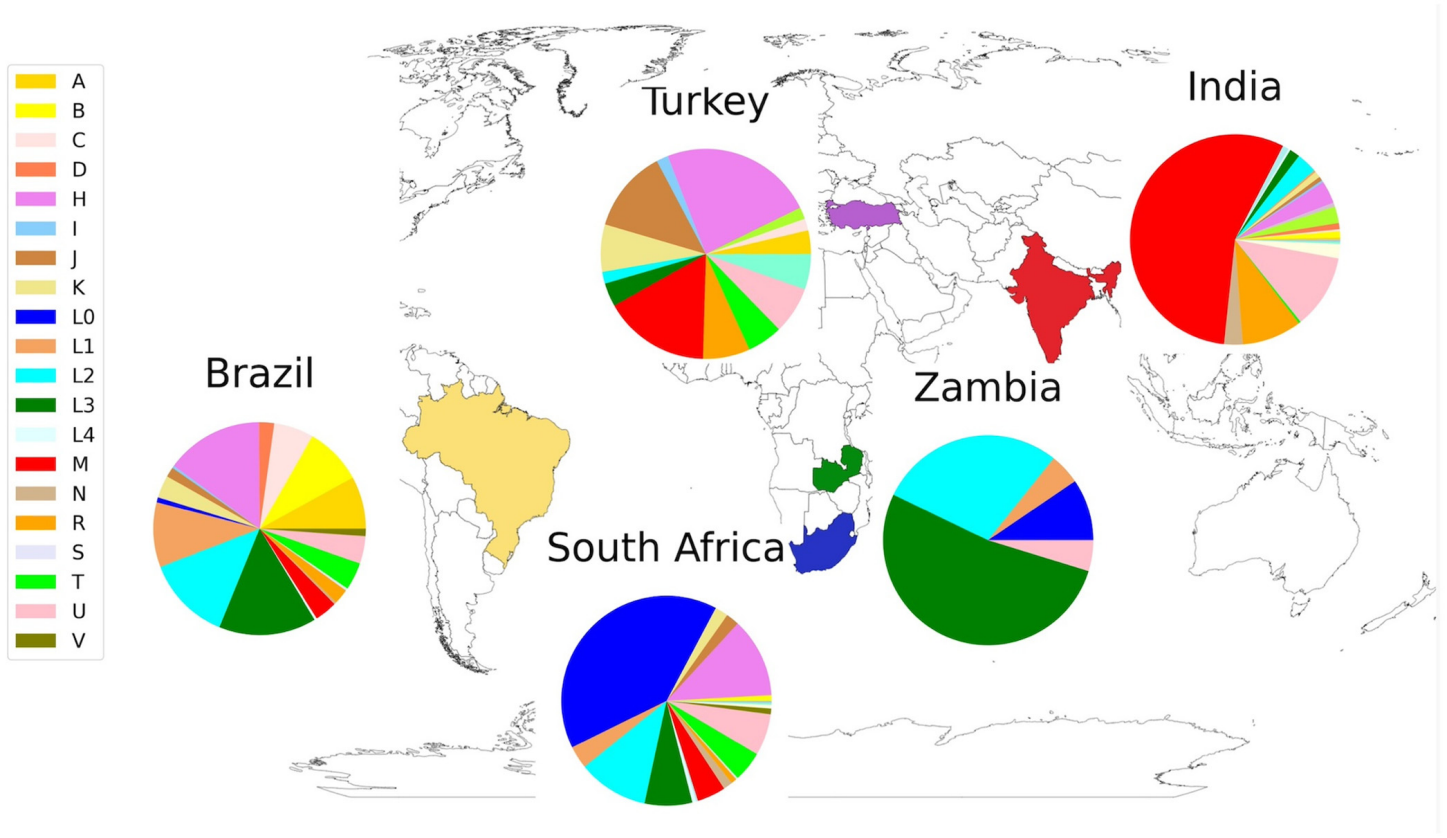
World map showing the countries which recruited study participants and pie charts showing the proportions of the different mitochondrial haplogroups identified in participants from each country. Brazil is shown in yellow, Turkey in purple, South Africa in blue, Zambia in green and India in red.

Following quality control steps, mtDNA variants were analysed for 998 individuals [589 males (59%), 409 females (41%), median age 25 years)] (Fig. 1a, b). The commonest diagnostic categories were genetic peripheral neuropathy (17.7%), limb‐girdle muscular dystrophy (17.5%) and congenital muscular dystrophy or myopathy (14.8%) (Fig. 1c). Approximately, 5.6% were recruited due to suspected mitochondrial disorders. Participants came from South Africa (342, 34%), India (315, 32%), Brazil (264, 26%), Turkey (55, 6%) and Zambia (21, 2%) (Fig. 2). Nine hundred and thirteen (91.5%) were probands, 28 (2.8%) were affected relatives and 57 (5.7%) were unaffected relatives. Singleton WES analysis of nuclear genes in part of this cohort reported a genetic diagnosis or strong candidate variant in 56% of 611 families.^14^ Clinical interpretation of the singleton WES results for the nuclear genes in the rest of the cohort is still ongoing.

### Sequencing depth

Median mtDNA sequencing depth was 28. Sequencing depth varied between individuals and between exome capture methods (Figure S1). Coverage was checked at positions of common pathogenic variants: sequencing depth was >10 in 86.7% of samples at position m.3243, 82.1% at m.8344 and 89.8% at m.8993. The m.3243A>G variant was not detected in the cohort, even in children with coverage >20 (Figure S2).

### Variants

A total of 33,614 variants were identified in 998 samples. After filtering, clinical interpretation was performed for 54 variants in 47 participants. Variants were reported to recruiting clinicians for 12 participants (including one participant who had two VUSs). There were two new diagnoses, two possible diagnoses and eight secondary findings (Table 1).

**Table 1:** Two confirmed diagnoses, two possible diagnoses and eight secondary findings of pathogenic variants with reduced penetrance.

Variant	Gene	HF%	ACMG Class	Country	Ethnicity	Sex	Haplogroup	Phenotype	AAO (years)
Confirmed diagnoses									
m.9035 T>C	MT‐ATP6	97	LP	South Africa	White	F	T	Spastic paraparesis, pes cavus, limb tremor, length‐dependent small fibre neuropathy, lower‐limb motor axonopathy, normal magnetic resonance imaging of brain and spinal cord. Multiple spontaneous abortions. Family history: affected brother.	20s
m.8993 T>G	MT‐ATP6	97	P	South Africa	Mixed‐African	F	U	Leigh syndrome with epilepsy and severe global developmental delay. No family history. Variant not detected in blood or urine in mother.	0 year 11 months
Possible diagnoses									
m.9984 G>A	MT‐CO3	23.5^a^	VUS	Turkey	Kurdish	M	J	Hypotonia in infancy, motor delay, speech delay, asymmetrical broad‐based gait. Fatigue. Magnetic resonance imaging showed patchy T2/flair hyperintense signal increase in bilateral periventricular white matter. Family history: mother has headaches and two spontaneous abortions. Variant 0% in blood, 1% in urine and buccal swab.	0
m.9026G>A m.12242A>G	MT‐ATP6 MT‐TS2	46 46	VUS VUS	South Africa	Mixed‐African	M	L0	Dysphagia, upgaze palsy, asymmetrical ptosis, diplopia and mildly elevated creatine kinase, type 2 diabetes mellitus Family history: dysphagia in two siblings, onset 60s	60
Secondary findings—deafness									
m.1494 C>T	MT‐RNR1	100	LP	India	Indian	F	M	Muscle weakness, ptosis and walking difficulties	2
m. 1555 A>G	MT‐RNR1	96.6	P	India	Indian	M	U	Limb girdle muscle weakness	12
m. 1555 A>G	MT‐RNR1	62.5	P	India	Indian	F	M	Upper‐limb muscle weakness, walking difficulty, headache, vomiting	29
m. 1555 A>G	MT‐RNR1	100	P	South Africa	Mixed‐African	F	L0	Distal muscle weakness and wasting with spasticity	1 year 10 months
m. 1555 A>G	MT‐RNR1	100	P	South Africa	Black African	M	L0	Generalised muscle weakness, facial weakness, ophthalmoparesis	0 year 0 month
Secondary findings—Leber hereditary optic neuropathy									
m.11778G>A	MT‐ND4	100	P	India	Indian	M	M	Hand muscle weakness and atrophy, fasciculations, polyminimyoclonus	39
m.14484 T>C	MT‐ND6	100	P	South Africa	Black African	F	L0	Rigidity, bradykinesia, motor regression, developmental regression, mask‐like facies	10
m.14484 T>C	MT‐ND6	100	P	South Africa	Black African	F	L0	Unaffected relative (mother of proband above)	‐

AAO, age at onset; HF, heteroplasmy fraction; LP, likely pathogenic; P, pathogenic, VUS, variant of uncertain significance.

^a^ Whole mtDNA analysis showed heteroplasmy 25% in blood, 22% in buccal swab DNA and 22% in urine DNA.

### Confirmed diagnoses

The m.9035T>C p.(Leu220Pro) variant in the *MT‐ATP6* gene has been reported in multiple families with spinocerebellar ataxia.^3^ The phenotype (spastic paraparesis and axonal neuropathy) and nearly homoplasmic level were consistent with previous literature.^18^ There was a family history of complicated hereditary spastic paraplegia in one brother.

The m.8993T>G; p.(Leu156Arg) variant in the *MT‐ATP6* gene is a commonly reported pathogenic variant which can cause Leigh syndrome (as seen in this patient) or Neuropathy Ataxia Retinitis Pigmentosa.^3^ Confirmatory testing by restriction fragment length polymorphism testing showed the variant was present at a near homoplasmic level in the proband. The variant was not detected in DNA extracted from blood or urine in the mother.

### Possible new diagnoses

A boy with hypotonia and developmental delay had the m.9984G>A p.(Gly260Ter) variant which affects the penultimate codon of the *MT‐CO3* gene. Whole mtDNA sequencing showed 25% heteroplasmy in blood and 22% in both buccal DNA and urine DNA. The variant was nearly absent in blood in the mother (1/544 reads) and present at 1% heteroplasmy in buccal and urine DNA. The heteroplasmy level in blood from the MToolBox pipeline (23.5%) was similar to the level measured by whole mtDNA sequencing (25%). The variant has been reported in two patients with childhood‐onset mitochondrial myopathy (heteroplasmy level not reported)^19^ and in a 15‐year‐old girl with intractable seizures^20^ (7.8% heteroplasmy in the blood). It is absent from the MitoMap and Helix population databases but presents as a heteroplasmic variant in six individuals in gnomAD v3.1. The residue has 100% conservation across the species in MitoMap.^16^

A participant from South Africa had two interesting mtDNA variants: m.9026G>A (p.Gly167Asp) in *MT‐APT6* and m.12242 A>G in the *MT‐TS2* gene (both 46% heteroplasmy). He has slowly progressive dysphagia (with onset >60 years), upgaze palsy, bilateral but asymmetrical ptosis and diplopia, glaucoma, mildly elevated creatine kinase and insulin‐dependent diabetes mellitus since age 50. He has two siblings with late‐onset dysphagia. Testing for oculopharyngeal muscular dystrophy 1 (*PABPN1* repeat expansion testing) and WES did not find a diagnosis. The m.9026G>A variant was reported at 87% heteroplasmy in an adult with ataxia and dystonic tremor^21^ and at lower heteroplasmy (~20%) in a child with intellectual disability, headaches, myalgia and fatigue.^3^ The residue shows 100% conservation across species. Five individuals in gnomAD v3.1 have the variant. The m.12242 A>G variant is absent from gnomAD. It has been published once in tumour and normal tissue of a patient with an oncocytic neoplasm.^22^ Conservation across species at the residue is relatively weak (77.8%).^16^ Although the m.9026G>A variant has been reported as disease causing,^3^, ^21^ it has reduced penetrance, and it is unclear whether it accounts for the participant's symptoms.

### Secondary findings—Pathogenic variants with reduced penetrance

Eight individuals had mtDNA variants which cause disorders with reduced penetrance. Five had variants in the *MT‐RNR1* gene which are associated with non‐syndromic or aminoglycoside‐induced deafness.^23^ None had a personal or family history of deafness.

Three had variants associated with LHON. None had a personal or family history of visual loss, although additional visual assessments were not performed. A girl with a paediatric onset movement disorder with choreiform movements and dystonic posturing of the hands and head, developmental regression and Parkinsonian symptoms had the m.14484T>C variant in the *MT‐ND6* gene. She was of Black African ethnicity (L0 haplogroup). She had no visual disturbance or optic atrophy. Due to the clinical presentation, the variant was assessed as unlikely to be causative.

### Haplogroups

In this study, 26/33 top‐level haplogroups were represented (Fig. 2, Table S1). Number of variants by country and haplogroup are shown in Figure S3.

## Discussion

Common pathogenic mtDNA variants found in people of European ancestry were very rare. There are several possible explanations. Firstly, clinicians in India, Brazil, South Africa and Turkey can source local mtDNA sequencing for individuals with typical symptoms. Secondly, it may reflect differences in genetic architecture between populations. The m.3243A>G variant has previously been reported in Indian patients with MELAS syndrome^24^ and in South African patients who had traditional Black African surnames.^8^ Thirdly, the ability to identify low‐level heteroplasmic variants is dependent on the number of sequencing reads that cover that position. A sequencing depth of 10 has been previously used for the detection of mtDNA variants with a heteroplasmy fraction of >10%.^11^ There was insufficient sequencing depth to reliably detect low‐level heteroplasmic variants. However, we did not observe m.3243A>G even in children (<15 years) with high (>20) sequencing depth at that position (Figure S2). The size of the cohort is also small compared to the prevalence of mitochondrial disorders, which has been estimated as 1 in 8000 in adults.^28^

Turkish participants were mainly of haplogroup H, so might be expected to have more of the common pathogenic mtDNA variants seen in people of European ancestry. A study of Leber Hereditary Optic Neuropathy in Turkish individuals showed that the three well‐known pathogenic variants were relatively rare (found in 5/32 individuals).^25^ A study of trio WES in 190 consanguineous Turkish families with neurogenetic disorders identified the m.3243A>G variant at a low level in a participant with an alternative genetic diagnosis and the m.14484T>C variant in one unaffected parent.^26^ Whole‐genome sequencing in 16 Turkish children with suspected mitochondrial disorders identified two definite diagnoses and two possible diagnoses, but the m.3243A>G variant was not observed.^27^

The diagnostic uplift was modest (2/998, 0.2%), comparable to previous studies. The diagnostic uplift was higher in participants recruited due to a suspected mitochondrial disorder (1/56, 1.8%). Poole et al^11^ found 11 diagnoses from 11,242 exomes (0.1%) in people with neurological disorders in the UK. Garret et al^12^ identified two diagnoses from 942 exomes (0.2%) in people with developmental and neurological disorders in France. Wagner et al^13^ reported a higher diagnostic yield, identifying 38 causative pathogenic variants in 2111 exomes (1.8%) in people with neurological disorders including 463 with suspected mitochondrial disorders in Germany.

Eight individuals had pathogenic homoplasmic variants that show reduced penetrance for deafness and LHON. There are ethical issues around whether these should be reported to participants. There is potential to prevent harm, for example, avoidance of aminoglycoside antibiotics but it could cause anxiety. There are currently no mtDNA genes on the American College of Medical Genetics and Genomics list for reporting in secondary findings.^29^ In the ICGNMD study, local policies for reporting were followed at each site.

A definite diagnosis and a possible diagnosis were made in people not clinically suspected to have a mitochondrial disorder, showing the utility of performing mtDNA analysis. Based on this study and our clinical experience, mtDNA analysis is especially useful in undiagnosed patients with peripheral neuropathy, spasticity or ataxia, in whom *MT‐ATP6* pathogenic variants may have been missed.

## Author Contributions

OYK, SR, KN, MS, NR, PT, DB, MKa, IP, KT, UY, BNN, DB, MKv, FH, SV, AN, CFRS, WM, SY, VVY, IS, JW, BC and JH contributed to data and sample acquisition. KT, UY, BNN, DB, MKv, FH, AN, WM, HT, MGH, SY, VVY, FvdW, IS, JW, BC, JH, PFC and RH contributed to study site leadership and management. FG, KRS, JV, SR, LAW, IS, SM, JW, BC, JH, PFC and RH contributed to data analysis and interpretation. FG, KRS, PFC and RH contributed to study concept and design. FG, KRS and RH drafted the text and figures.

## Conflict of Interest

The authors declared no conflict of interest.

## Supporting information


**Figure S1.** Overall mtDNA coverage by centre. The *x*‐axis shows the position along the mitochondrial chromosome and the *y*‐axis shows the depth of coverage. Each coloured line represents an individual participant. Two distinct patterns of coverage can be observed. South Africa, Zambia and Brazil used the same sequencing provider and show similar patterns of coverage.
**Figure S2.** Scatterplot showing sequencing depth at position m.3243 and age at recruitment. The x‐axis shows the age at recruitment and the *y*‐axis shows sequencing depth at position m.3243. The dots represent individual participants. The red horizontal line is at sequencing depth 20 and the green vertical line is at age 15 years. There were 231 children aged <15 years with sequencing depth >20, 85 children aged <15 years with sequencing depth ≤20, 407 individuals aged ≥15 years with sequencing depth >20 and 275 individuals aged ≥15 years with sequencing depth ≤20.
**Figure S3.** Number of variants were identified in individuals from different countries. The *y*‐axis shows the total number of variants found in an individual. Individual results are shown with dots which are coloured according to the individual's haplogroup. Across different centres, high number of variants are observed in people with L0 and L1 haplogroups.
**Table S1.** Numbers of individuals with each haplogroup by country.

## Data Availability

At the end of the study, participants de‐identified exome and genome data will be archived in the European Molecular Biology Laboratory European Bioinformatics Institute's European Genome‐Phenome Archive (EMBL EBI EGA), with community access to this and selected de‐identified REDCap data managed via an ICGNMD Data Access Committee.
