# Supplementary material for: Mitochondrial DNA disorders in neuromuscular diseases in diverse populations
Source: Ann Clin Transl Neurol. 2024 Aug 2;12(8):1680–8. doi: 10.1002/acn3.52141 (PMC12343313; doi:10.1002/acn3.52141)
Supplement: Supplementary file 1 — Figure S1. Overall mtDNA coverage by centre. The x‐axis shows the position along the mitochondrial chromosome and the y‐axis shows the depth of coverage. Each coloured line represents an individual participant. Two distinct patterns of coverage can be observed. South Africa, Zambia and Brazil used the same sequencing provider and show similar patterns of coverage. Figure S2. Scatterplot showing sequencing depth at position m.3243 and age at recruitment. The x‐axis shows the age at recruitment and the y‐axis shows sequencing depth at position m.3243. The dots represent individual participants. The red horizontal line is at sequencing depth 20 and the green vertical line is at age 15 years. There were 231 children aged <15 years with sequencing depth >20, 85 children aged <15 years with sequencing depth ≤20, 407 individuals aged ≥15 years with sequencing depth >20 and 275 individuals aged ≥15 years with sequencing depth ≤20. Figure S3. Number of variants were identified in individuals from different countries. The y‐axis shows the total number of variants found in an individual. Individual results are shown with dots which are coloured according to the individual's haplogroup. Across different centres, high number of variants are observed in people with L0 and L1 haplogroups. Table S1. Numbers of individuals with each haplogroup by country. [file ACN3-12-1680-s001.docx]

**Supplementary Methods**

**Library generation for whole exome sequencing**

*South Africa, Brazil and Zambia*

Libraries were generated at Macrogen Europe (Amsterdam, The Netherlands) using SureSelectXT V6 panel (Agilent).

*Turkey*

Libraries were generated in Ankara using QIAseq Human Exome (QUIAGEN).

*India*

Libraries were generated at CCMB (Hyderabad, India) using either TruSeq DNA exome (Illumina) or Twist whole exome sequencing (Twist Bioscience), or at MedGenome (Bangalore, India) using Twist whole exome sequencing (Twist Bioscience).


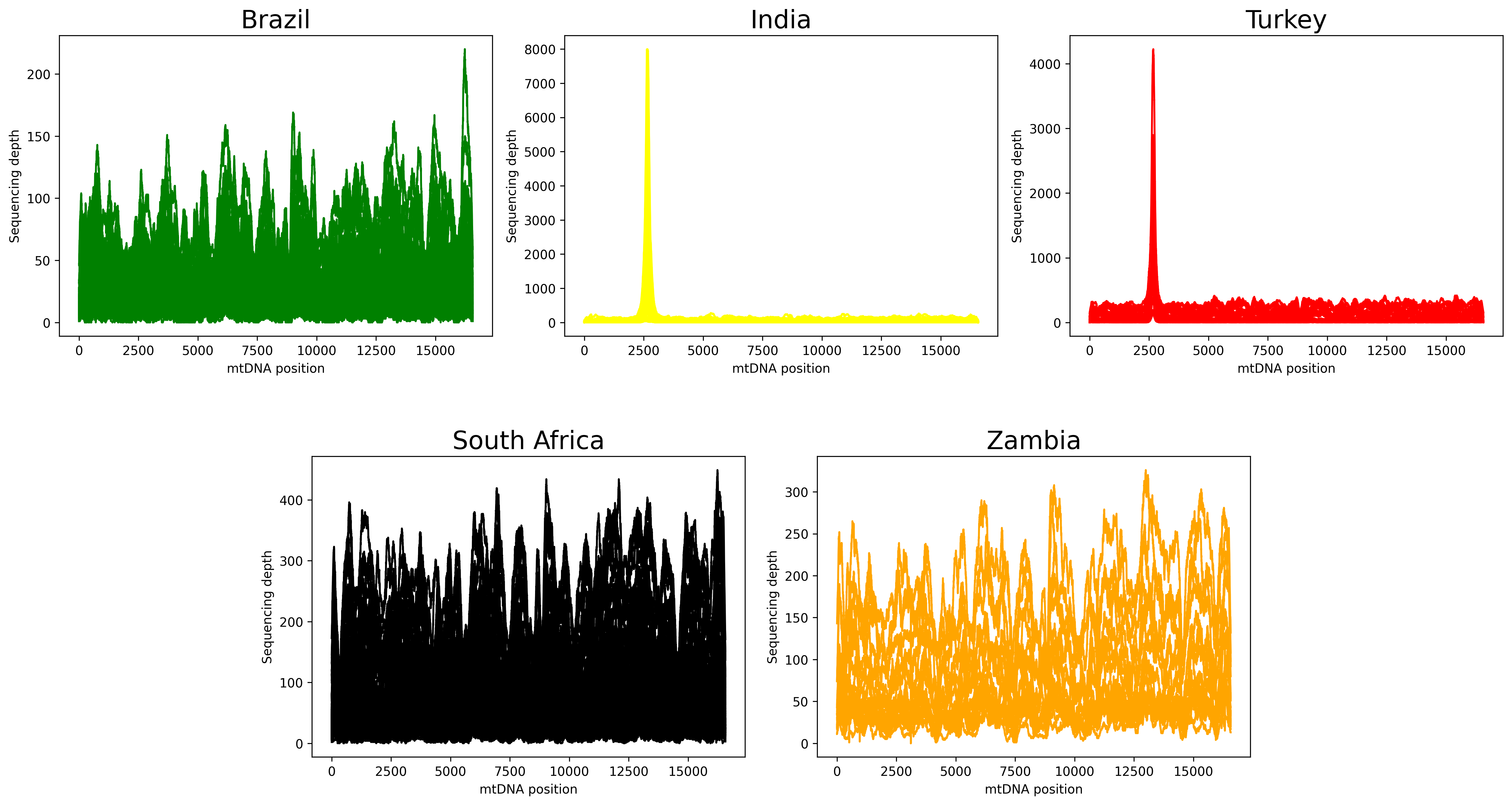


**Supplementary Figure 1** Overall mtDNA coverage by centre. The x-axis shows the position along the mitochondrial chromosome and the y-axis shows the depth of coverage. Each coloured line represents an individual participant. Two distinct patterns of coverage can be observed. South Africa, Zambia and Brazil used the same sequencing provider and show similar patterns of coverage. We are uncertain about the reason for the peak observed at around position m.2500 in the Indian and Turkish data, but it may relate to the library preparation used.

**
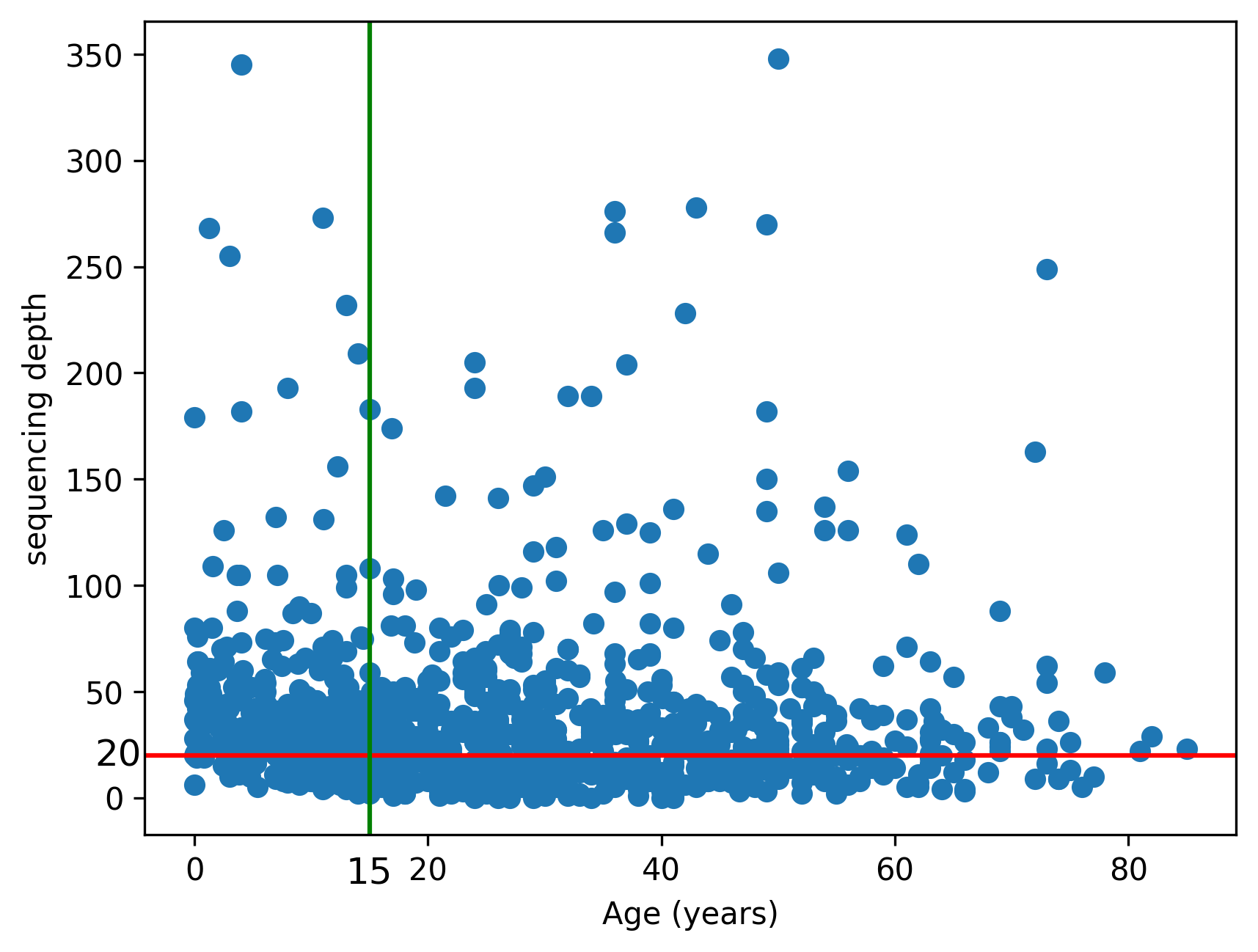
**

**Supplementary Figure 2** Scatterplot showing sequencing depth at position m.3243 and age at recruitment. The x-axis shows the age at recruitment and the y-axis shows sequencing depth at position m.3243. The dots represent individual participants. The red horizontal line is at sequencing depth 20 and the green vertical line is at age 15 years. There were 231 children aged <15 years with sequencing depth >20, 85 children aged <15 years with sequencing depth ≤ 20, 407 individuals aged ≥15 years with sequencing depth >20 and 275 individuals aged ≥15 years with sequencing depth ≤ 20.


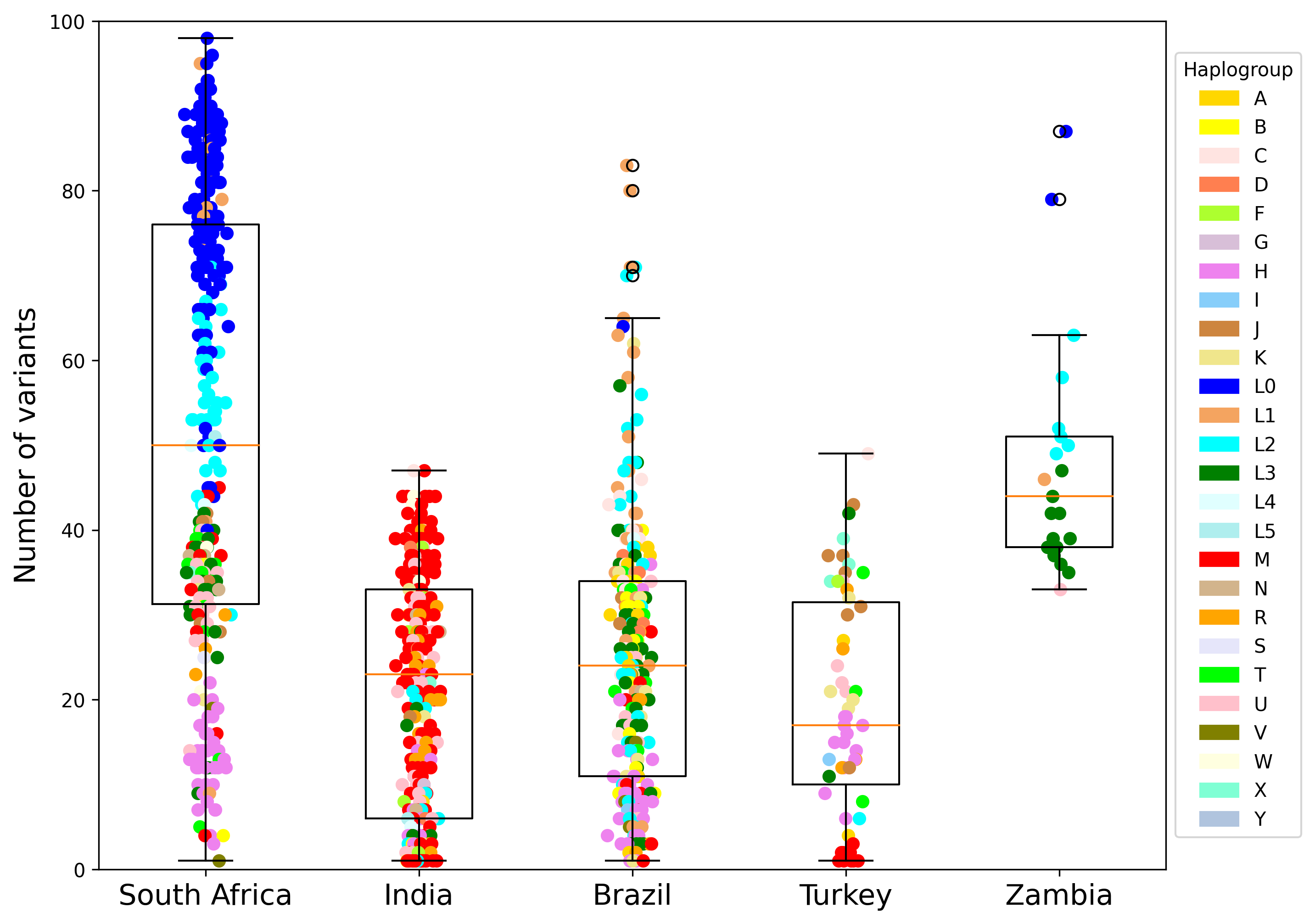


**Supplementary Figure 3** Numbers of variants identified in individuals from different countries. The y-axis shows the total number of variants found in an individual. Individual results are shown with dots which are coloured according to the individual’s haplogroup. Across different centres, high numbers of variants are observed in people with L0 and L1 haplogroups

**Supplementary Tables** Numbers of individuals with each haplogroup by country

**Brazil**

| **Haplogroup** | **Number of participants** | **Percentage** |
| --- | --- | --- |
| H | 39 | 14.8% |
| L3 | 39 | 14.8% |
| L2 | 34 | 12.9% |
| L1 | 26 | 9.9% |
| B | 23 | 8.7% |
| A | 21 | 8.0% |
| C | 16 | 6.1% |
| T | 11 | 4.2% |
| U | 11 | 4.2% |
| K | 9 | 3.4% |
| M | 9 | 3.4% |
| D | 6 | 2.3% |
| R | 6 | 2.3% |
| J | 4 | 1.5% |
| V | 3 | 1.1% |
| L0 | 2 | 0.8% |
| I | 1 | 0.4% |
| L4 | 1 | 0.4% |
| N | 1 | 0.4% |
| S | 1 | 0.4% |

**South Africa**

| **Haplogroup** | **Number of participants** | **Percentage** |
| --- | --- | --- |
| L0 | 137 | 40.1% |
| H | 42 | 12.3% |
| L2 | 37 | 10.8% |
| L3 | 25 | 7.3% |
| U | 22 | 6.4% |
| T | 16 | 4.7% |
| M | 15 | 4.4% |
| L1 | 12 | 3.5% |
| J | 7 | 2.0% |
| K | 7 | 2.0% |
| N | 5 | 1.5% |
| B | 3 | 0.9% |
| R | 3 | 0.9% |
| V | 3 | 0.9% |
| L4 | 2 | 0.6% |
| W | 2 | 0.6% |
| L5 | 1 | 0.3% |
| S | 1 | 0.3% |
| X | 1 | 0.3% |
| Y | 1 | 0.3% |

**India**

| **Haplogroup** | **Number of participants** | **Percentage** |
| --- | --- | --- |
| M | 176 | 55.9% |
| U | 36 | 11.4% |
| R | 29 | 9.2% |
| H | 11 | 3.5% |
| L2 | 10 | 3.2% |
| N | 9 | 2.9% |
| F | 8 | 2.5% |
| W | 7 | 2.2% |
| L3 | 5 | 1.6% |
| B | 3 | 1.0% |
| D | 3 | 1.0% |
| K | 3 | 1.0% |
| L5 | 3 | 1.0% |
| G | 2 | 0.6% |
| J | 2 | 0.6% |
| A | 1 | 0.3% |
| C | 1 | 0.3% |
| I | 1 | 0.3% |
| L1 | 1 | 0.3% |
| L4 | 1 | 0.3% |
| T | 1 | 0.3% |
| X | 1 | 0.3% |
| Y | 1 | 0.3% |

**Turkey**

| **Haplogroup** | **Number of Participants** | **Percentage** |
| --- | --- | --- |
| H | 13 | 23.6% |
| M | 9 | 16.4% |
| J | 7 | 12.7% |
| K | 4 | 7.3% |
| R | 4 | 7.3% |
| U | 4 | 7.3% |
| T | 3 | 5.5% |
| X | 3 | 5.5% |
| A | 2 | 3.6% |
| L3 | 2 | 3.6% |
| C | 1 | 1.8% |
| F | 1 | 1.8% |
| I | 1 | 1.8% |
| L2 | 1 | 1.8% |

**Zambia**

| **Haplogroup** | **Number of participants** | **Percentage** |
| --- | --- | --- |
| L3 | 11 | 52.4% |
| L2 | 6 | 28.6% |
| L0 | 2 | 9.5% |
| L1 | 1 | 4.8% |
| U | 1 | 4.8% |
